# Surgical antibiotic prophylaxis in children: a mixed method study on healthcare professionals attitudes

**DOI:** 10.1186/s12887-016-0739-y

**Published:** 2016-12-05

**Authors:** Angela Giusti, Stefania Spila Alegiani, Marta Luisa Ciofi degli Atti, Sofia Colaceci, Roberto Raschetti, Pasquale Arace, Raffaele Spiazzi, Massimiliano Raponi, The Apache Study Group

**Affiliations:** 1 National Centre for Epidemiology, Surveillance and Health Promotion, National Institute of Health, Rome, Italy; 2 Clinical Epidemiology Unit, Medical Direction, Bambino Gesù Children’s Hospital, Rome, Italy; 3 Medical Direction, Ospedale Santobono Pausilipon, Naples, Italy; 4 Medical Direction, Children’s Hospital AO Spedali Civili, Brescia, Italy; 5 Medical Direction, Bambino Gesù Children’s Hospital, Rome, Italy

**Keywords:** Antibiotic prophylaxis, Qualitative research, Attitude of health personnel, Children

## Abstract

**Background:**

Qualitative and quantitative research investigating determinants of adherence to clinical guidelines (GLs) on surgical antibiotic prophylaxis (SAP) are scarce. We conducted a mixed-method study aimed at investigating barriers and at describing attitudes of healthcare professionals (HCPs) regarding SAP in three Italian children’s hospitals.

**Methods:**

The study comprised two sequential phases: 1) collection of qualitative data through focus groups; 2) conduction of a survey on HCPs attitudes towards SAP. Focus groups were carried out in each hospital with a theoretical convenience sample of 10–15 HCPs. Categorical analysis was conducted. Emerging categories and additional topics derived by literature search were used to develop the survey questionnaire, which included 13 questions expressed through a 4-point Likert scale. Members of surgical teams were invited by e-mail to fill in the questionnaire. We summed up the points assigned to each 4-point Likert scale response and calculated a cumulative score expressing overall concordance to expected HCPs attitudes on SAP. We conducted univariate and multivariate analysis to evaluate the relationship among characteristics of respondents and concordance with expected attitudes.

**Results:**

The main categories identified in the qualitative phase included determinants of general adherence to GLs (e.g., relevance of clinical judgment), individual determinants (e.g., poor knowledge on hospital data) and organizational/structural determinants (e.g., patient flows). A total of 357 HCPs participated in the survey (response rate: 82.1%). Among respondents, 75% reported that SAP should be performed with first or second-generation cephalosporins, 44% that 2–3 days of antibiotic administration are useful as a precaution after surgery, 32% that SAP is needed for all surgical procedures. At multivariate analysis, professional category (physicians vs nurses; OR: 3.31; 95%CI: 1.88–5.82), and hospital (hospital 1 and 2 vs hospital 3; ORs: 2.79, 95%CI: 1.22–6.36; 2.40, 95%CI: 1.30–4.43, respectively) were significantly and independently associated with higher concordance with expected attitudes on SAP.

**Conclusions:**

Results from this study were useful to identify obstacles to appropriate SAP use in children. In our setting, findings support that a quality-improvement intervention should take into account local contexts, with development of hospital policies, education on SAP recommendations, and dissemination of data on adherence to recommendations.

## Background

Surgical site infections (SSIs) are one of the most common complications following surgery and are responsible for an increase in postoperative morbidity and mortality and healthcare-associated costs [1]. The effectiveness of surgical antibiotic prophylaxis (SAP) in the prevention of SSIs was established in the early 1960s [2]. Current clinical guidelines (GLs) [3, 4] define procedures requiring SAP, recommend that SAP should be administered as a single dose, with the exception of special circumstances (such as prolonged surgery or major blood loss), and narrow spectrum, less expensive antibiotics (e.g. first or second-generation cephalosporins) should be the first choice. The quality of SAP has been the subject of many audits [5, 6] and intervention studies [7–9]. However, observance of GLs is often suboptimal, both in adults and children [3, 10–12]. Translation of evidence-based recommendations into clinical practice is notoriously challenging. Lack of awareness, lack of familiarity, lack of agreement, lack of self-efficacy, lack of outcome expectancy, the inertia of previous practices, and external barriers are described as obstacles to adherence of physician to GLs [13]. Qualitative and quantitative studies investigating determinants of adherence to clinical guidelines on SAP are scarce. In a single study on adults, obstacles to the observance of recommendations on proper SAP timing included low priority, inconvenience, workflow, organizational communication, and role perception [14]. In 2012, the Italian Agency of Drugs funded a multicenter project to promote appropriateness of SAP in children [15], which included a mixed-method study, aimed at investigating barriers and at describing attitudes of healthcare professionals (HCPs) regarding SAP in three Italian children’s hospitals, in order to tailor strategies for improving adherence to recommendations.

## Methods

This study was conducted in three tertiary care children’s hospitals, located in northern (Children’s Hospital AO Spedali Civili, Brescia; 192 inpatient beds; Hospital 1), central (Bambino Gesù Children’s Hospital, Rome; 607 beds, Hospital 2) and southern Italy (Ospedale Santobono Pausilipon, Naples; 442 beds, Hospital 3). These hospitals included the largest children’s hospitals in Italy (Bambino Gesù Children’s Hospital) and a convenience sample of two other children’s hospitals located throughout Italy. At the time of the study, two hospitals (Hospital 1 and 3) had local GLs on SAP, developed by hospital multidisciplinary groups.

The study took place between June 2012 and February 2013; it had a mixed-method exploratory design and comprised two sequential phases [16], i.e. 1) collection of qualitative data through focus groups on general adherence to GLs and adherence to SAP recommendations; 2) conduction of a survey on the HCPs attitude towards SAP.

The study received approval by the Ethical Committees of the three participating hospitals. All collected data were anonymous and confidential.

### Phase one: qualitative study

Between June and July, 2012, three focus groups were carried out in the participating hospitals.

In each hospital, the local research investigators directly invited 10 to 15 HCPs involved in prescription and administration of SAP to participate in the focus group, including at least one representative of hospital anesthesiologists, surgeons and nurse coordinators from operating rooms and surgical wards. Participants were recruited through a theoretical convenience sample. The theoretical sampling aims to include as many as possible of the factors that might affect variability of behavior, and then this is extended, as required, in the light of early findings and emergent theory [17]. In our study, all key responders were included in the initial sampling and no further sampling was needed. In addition to being theory driven, our sample was a convenience sample as the key responders were selected among others, based on their willingness to participate to the focus groups.

The focus groups were conducted by a study researcher following a grid of semi-structured questions to explore the participants’ perception regarding determinants of the individual SAP behaviour (Table 1). Each participant reported socio-demographic data on an anonymous, self-administered form. All focus groups were digitally audio-recorded and fully transcribed. All participants provided informed consent.

**Table 1:** Grid of semi-structured questions to explore healthcare professionals’ perception on determinants of SAP behavior

Aim	Question
Attitude and practice underlying SAP behaviour	1. What is your opinion about the surgical antibiotic prophylaxis in your hospital? 2. According to clinical guidelines, the use of third or fourth-generation cephalosporins is not recommended for surgical prophylaxis. In fact, they have not been proven to be more effective than first or second-generation cephalosporins, while there are many negative effects caused by the inappropriate use of these antibiotics, including the emergence of antibiotic resistance. Another recommendation concerns the duration of antibiotic prophylaxis, that should be limited to the perioperative period, while the decision to continue prophylaxis beyond the first 24 postoperative hours is not recommended. a. What is your opinion? b. What are, in your opinion, the obstacles to the application of these recommendations?

Categories were developed both deductively, based on the research questions, and inductively, based on new contents emerging from the data. Thus, text transcriptions were coded according to the pre-defined categories derived from the interviews’ questions and new categories were built on the basis of newly emerging topics. Saturation of contents was reached when no new category emerged from the data [18]. Coding, content analysis and mapping were carried out using NVivo10 and Free MindMap.

### Phase two: quantitative study

Some of the categories emerging from the qualitative study and additional topics derived from the literature on antibiotic prescriptions [13, 19, 20] were used to develop a questionnaire on HCP attitudes.

The self-administered questionnaire included the following items: HCP characteristics (hospital, age, sex, professional category, length of practice), one question on sources of information on SAP (hospital guidelines, national guidelines, international guidelines or personal experience; maximum of two responses allowed), and 13 questions on attitudes regarding SAP expressed through a 4-point Likert scale (“completely disagree”, “disagree”, “agree”, and “completely agree”).

In February 2013, members of surgical teams who participated in the above mentioned multicenter project on SAP use [15] were invited by e-mail to fill in the questionnaire on a web-platform. Non-respondents were contacted personally and completed the questionnaire on paper. Data entry of paper questionnaires was centralized.

Continuous variables were described as mean values ± standard deviation and analyzed using a two-sided Student’s test if they were normally distributed or a two-sided Wilcoxon rank-sum test if they were not. Categorical variables were described as proportions and compared using the Chi-squared or Fisher’s test, as appropriate. The 4-point Likert scale responses were scored as follows “1-completely concordant with expected attitudes”, “2- concordant”, “3-not concordant”, “4-completely not concordant”. We calculated a cumulative score by summing up the points assigned to each response. The cumulative score ranged from 13 (highest concordance with expected attitudes) to 52 (lowest concordance with expected attitudes). At the univariate analysis, we compared results by characteristics of respondents. The level of significance was set at a *p*-value = 0.05. We conducted a multivariate logistic regression analysis to evaluate the relationship between characteristics of respondents and concordance with expected attitudes on SAP. SPSS software (IBM SPSS statistics, version 22) was used for the statistical analyses.

## Results

### Qualitative results

Thirty-three HCPs participated in the focus groups, including surgeons (N. 15), nurse coordinators (N. 10), and anesthesiologists (N. 8). The participants’ mean age was 49.1 years (range: 31–60); 18 were females (55%), and the mean length of practice in pediatric surgery was 18.2 years (range: 0–34).

**Fig. 1:**
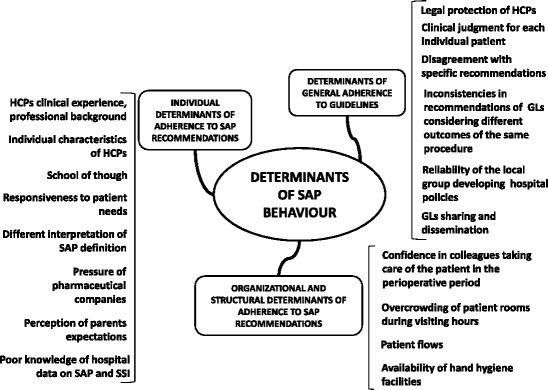
Determinants of the individual SAP behavior. Map of the emerging categories

Within the data emerging from the area of determinants of the SAP behavior, 3 main categories were identified, both deductively and inductively (Fig. 1):

1. Determinants of general adherence to GLs
2. Individual determinants of adherence to SAP recommendations
3. Organizational and structural determinants of adherence to SAP recommendations.

#### Determinants of general adherence to GLs

Barriers to adherence to SAP GLs included disagreement of HCPs with specific recommendations, such as antibiotic choice, due to the perception of lower efficacy of first or second-generation cephalosporins compared to third-generation cephalosporins.

The GLs were well accepted by HCPs if shared and communicated appropriately. HCPs underlined the need of clinical judgment, since recommendations should be tailored to each patient, taking into account clinical conditions.

Wherever hospital GLs were available, HCPs trusted the group that developed policies and procedures, in particular if it was a multidisciplinary group of peers with recognized scientific and methodological knowledge and experience of the local context.

GLs were perceived as a protective tool in case of litigations for medical malpractice, although they would not exempt HCPs from clinical judgment.

*“… The fact that there are people who study the literature, that maybe you do not have time to see and interpret, then those people are our people* (Editor’s Note-Ed. colleagues from the same hospital), *people who work like us”*

*“… In a sense* (Ed. hospital GLs) *do not legally safeguard you, however they* (Ed. colleagues who developed hospital GLs) *had more time than me to study this issue, so I do not know it but I accept it, provided that if I think I had to change* (Ed. SAP prescription on each individual patient) *I can change it”*

#### Individual determinants of adherence to SAP recommendations

Individual determinants included: type of practice, professional background and clinical experience, responsiveness to patients’ needs, willingness to *“do what is right”* and hospital’s “*school of thought”*. Individual characteristics such as the ability to manage anxiety and stress were also mentioned.

Barriers in this category included the expectation of parents regarding SAP, though it was reported that families generally prefer to avoid prolonged administration of antibiotics, and the pressure of pharmaceutical companies, that could influence the choice of antibiotics, deviating from recommendations.

Other barriers were poor knowledge of hospital data on quality of SAP administration and incidence of surgical site infections.

*“We have no idea of our numbers, regarding the type and volumes of surgical procedures, the type and mode of administration of SAP we practice, our incidence of hospital acquired infections, either surgical site infections, or bacteremia etc. From what, for example, I read, it seems to me that our data are even lower than those found in the literature, because the literature sometimes reports huge numbers. But this is just a feeling, because I really do not know* (Ed. hospital data)”.

The meaning of surgical “prophylaxis” was subject to different interpretations, since the definition of SAP as administration of antibiotics prior to surgical incision to prevent SSI was not uniformly agreed. The expression “prolonged prophylaxis”, i.e. the administration of antibiotics as a precautionary measure over a period longer than recommended, or in patients not at risk, was also mentioned.

#### Organizational and structural determinants of adherence to SAP recommendations

Factors which may influence adherence to SAP included confidence in colleagues who take care of the patient in the perioperative period. In detail, failures in timing of administration, adherence to asepsis during procedures, and post-operative management of the surgical site may cause an over-use of SAP, either in terms of administering antibiotics in procedures where SAP is not indicated, choosing a second choice antibiotic, or administering antibiotics for more than 24 h.

Overcrowding of patients’ rooms due to the presence of many relatives during visiting hours, proximity to other potentially infectious patients, absence of clean patient routes (e.g. elevators restricted for the operating room), or of hand hygiene facilities in every room may also cause overuse of SAP.

### Quantitative results

Out of 435 questionnaires sent to HCPs, 357 were returned (244 filled out the questionnaire on the web-platform and 113 on paper), resulting in a response rate of 82.1%.

Response rates and characteristics of respondents by hospital are reported in Table 2. Survey respondents were mainly physicians (56.3%) and were evenly distributed by sex. Their median age was 49.0 years and the median length of practice was 19 years. Age and length of practice were directly related and significantly differed by hospital.

**Table 2:** Characteristics of respondents to the questionnaire on SAP attitudes, by participating hospital

	Hospital 1	Hospital 2	Hospital 3	Total	*p*-value
Number of sent questionnaire	59	273	103	435	
Number (%) of returned questionnaires	54 (91.5)	223 (81.7)	80 (77.7)	357 (82.1)	
Characteristics of respondents					
Median age (range), years^a^	45 (27–63)	46 (27–68)	53 (32–72)	49 (27–72)	<0.01
Sex					
Males (%)	22 (40.7)	108 (48.4)	47 (58.8)	177 (49.6)	
Females (%)	32 (59.3)	115 (51.6)	33 (41.2)	180 (50.4)	
Healthcare professional					
Physicians (%)	27 (50.0)	128 (57.4)	46 (57.5)	201 (56.3)	
Nurses (%)	27 (50.0)	95 (42.6)	34 (42.5)	156 (47.7)	
Median lenght of practice (range), years^b^	15 (2–39)	15 (1–40)	24 (1–44)	19 (1–44)	<0.05

^a^missing data for 9 HCPs

^b^missing data for 10 HCPs

Seventy-five percent of HCPs (266/357) used hospital guidelines as reference for SAP, 50% (N. 180) used international guidelines, and 25% (N. 89) national guidelines. Only 8% (N. 29) of respondents choose SAP on the basis of their personal experience. The proportion of HCPs referring to their personal experience was related to length of practice (4.1% if length of practice was <18 years and 11.8% if length of practice was ≥18 years, *p* < 0.05) and hospital (4.3% in the two centres with local GLs, 21.3% in the hospital were local GLs were not implemented, *p* < 0.01).

Table 3 reports the results regarding questions on SAP attitudes. The majority of respondents (≥75%) agreed or completely agreed on the following items: their hospital took into account international GLs on SAP; GLs could be supportive in case of malpractice litigations; SAP should be performed with first or second-generation cephalosporins.

**Table 3:** Attitudes on SAP by responses to 4-point Likert scale questions (N. 357)

	completely disagree		disagree		agree		completely agree		No response	
	N.	%	N.	%	N.	%	N.	%	N.	%
1. The hospital where I work takes into account the international GLs on SAP	3	0.8	38	10.6	238	66.7	75	21.0	3	0.8
2. SAP should be performed with first or second-generation cephalosporins	62	17.4	10	2.8	47	13.2	220	61.6	18	5.0
3. Two to 3 days of antibiotic administration are useful as a precaution after surgery	49	13.7	145	40.6	127	35.6	29	8.1	7	2.0
4. Prescription of SAP should take into account possible malpractice litigations	31	8.7	148	41.5	149	41.7	20	5.6	9	2.5
5. GLs may be supportive in case of malpractice litigations	20	5.6	2	0.6	61	17.1	270	75.6	4	1.1
6. Evidence-Based Medicine is poorly applicable in every day clinical practice	34	9.5	203	56.9	105	29.4	7	2.0	8	2.2
7. SAP duration should take into account parental expectations	134	37.5	203	56.9	15	4.2	3	0.8	2	0.6
8. SAP is needed in all surgical procedures	54	15.1	189	52.9	89	24.9	25	7.0	0	0.0
9. The threat of antibiotic resistance is overstated by the media	58	16.2	213	59.7	71	19.9	9	2.5	6	1.7
10. Antibiotic resistance concern patients different from the children cared by the Hospital where I work	80	22.4	233	65.3	34	9.5	3	0.8	7	2.0
11. The choice of drug for SAP is mainly due to cost-saving reasons	53	14.8	221	61.9	67	18.8	6	1.7	10	2.8
12. First or second-generation cephalosporins are less effective for SAP than III generation cephalosporins	42	11.8	229	64.1	52	14.6	4	1.1	30	8.4
13. Pharmaceutical companies influence antibiotics used for SAP	65	18.2	183	51.3	82	23.0	15	4.2	12	3.4

*N.* number

A proportion of HCPs ranging from 30 to 50% agreed on the following items: Evidence-Based Medicine is poorly applicable in every day clinical practice; SAP is needed for all surgical procedures; 2–3 days of antibiotic administration are useful as a precaution after surgery; prescription of SAP should take into account possible malpractice litigations.

Finally, less than 30% of respondents agreed on the following items: first or second-generation cephalosporins are less effective than third-generation cephalosporins; the threat of antibiotic resistance is overstated by the media; antibiotic resistance does not concern children they care for; the choice of drug for SAP is mainly due to cost-saving reasons; SAP duration should consider parental expectations; pharmaceutical companies influence antibiotics used for SAP.

The cumulative score of responses was calculated over 301 questionnaires that had been fully completed. The median cumulative score was 27 (interquartile range 25–30), over a scale from 13 to 52. Table 4 shows determinants of concordance with expected attitudes on SAP, by univariate and multivariate analysis. At the univariate analysis, professional category (physicians vs nurses), length of practice (≥18 years vs <18) and hospital (hospital 1 and 2 vs hospital 3) were significantly associated with the proportion of respondents whose cumulative score was <27, i.e. had a higher concordance with expected attitudes. At the multivariate analysis, professional category and hospital remained significantly and independently associated with cumulative score value.

**Table 4:** Determinants of concordance with expected attitudes on SAP, by univariate and multivariate analysis (concordance expressed as cumulative score; lower values of the score indicate higher concordance with expected attitudes) (N. 301)

	N.	Cumulative score < 27	Univariate analysis		Multivariate analysis	
			OR	95% CI	OR	95% CI
Sex						
Females	141	48%	1		1	
Males	160	54%	1.28	0.81–2.01	0.86	0.50–1.48
Health care professional						
Nurses	115	35%	1		1	
Physicians	186	62%	3.04	1.87–4.93	3.31	1.88–5.82
Length of practice						
≥ 18 years	147	46%	1		1	
< 18 years	147	57%	1.59	1.00–2.52	1.22	0.74–1.99
Hospital						
Hospital 3	68	34%	1		1	
Hospital 1	45	58%	2.68	1.23–5.82	2.79	1.22–6.36
Hospital 2	188	56%	2.53	1.42–4.51	2.40	1.30–4.43

*N.* number

*OR* Odds Ratio

*CI* Confidence Interval

## Discussion

This mixed method study conducted in three tertiary care hospitals allowed to investigate the attitude of HCPs regarding SAP in children from different perspectives.

There was convergence between qualitative and quantitative results in considering clinical guidelines as reliable tools, safeguarding clinicians in case of malpractice litigation. Willingness to adhere to GLs was very high, with only 9% of HCPs who participated in the quantitative survey stating that they based their SAP prescription on their personal experience rather than on GLs. These latter HCPs had a significantly higher length of practice compared to colleagues that did not quote personal experience as a source of information for SAP administration. This finding confirms results from a study on antimicrobial prescriptions in Danish hospitals, that showed how younger professionals have a higher confidence in GLs than older HCPs [21]. However, according to our results, the availability of hospital GLs is also a determinant of source of information on SAP, since only 4.3% of HCPs quoted their personal experience as a source of information in the two centers with local GLs, compared to 21.3% in the hospital were local GLs were not implemented.

The available evidence suggests that audit and feedback may be effective in improving professional practice [22–24]. The need of data feedback on process and outcome indicators was clear from qualitative results, since HCPs complained about the poor availability of local data on SAP characteristics and on outcomes of surgical procedures, including surgical site infections.

Previous studies have shown that antibiotic choice and duration of administration are critical areas for adherence to GL recommendations on SAP [6, 11, 25]. A prospective investigation of SAP characteristics, carried out in the same three hospitals that took part in the present study, showed that third or fourth-generation cephalosporins, carbapenems or piperacillin/tazobactam were used in 19% of procedures and duration was longer than 24 h in 84% [15].

In our results, there was convergence on the knowledge that first or second-generation cephalosporins should be used and that they are as effective as third or fourth-generation cephalosporins for SAP. Financial concerns and pressures by pharmaceutical company were not perceived as strongly relevant in the choice of drugs to be used for SAP. Nevertheless, some focus group participants suggested that a part of the hospital’s personnel might not be aware of the appropriate choice, as confirmed by the finding that 20% of respondents to the quantitative study do not agreed that SAP should be performed with first or second-generation cephalosporins.

Regarding duration of prophylaxis, a single pre-operative dose is recommended for most surgical procedures. The prolonged use of antimicrobials after this period does not provide additional benefits and is associated with increased risk of adverse events and induction of antimicrobial resistance [26]. Participants to this study showed a sound perception of the issue of antimicrobial resistance, as confirmed by qualitative and quantitative results. However, in line with results on the adherence to SAP duration [15], there was a misconception among many HCPs regarding the duration of SAP, described in the focus groups as “prolonged prophylaxis”. Quantitative results showed 44% of agreement regarding the use of antibiotic treatment during 2–3 days after the surgical procedure, as a precautionary approach.

Parental expectation was described in the focus group both as excessive and as defective: according to the participants’ opinion, some parents seem more keen regarding a precautionary overuse of antibiotics after a surgical procedure, while others prefer not to use antibiotics at all. In any case, only 5% of responders to quantitative survey considered that parental expectation should drive duration of SAP. This finding shows that while parental pressure and expectations is a strong determinant in primary setting [19, 27], it may be less relevant in the hospital surgical setting.

The results of the multivariate analysis showed that professional category and hospital were independent predictors of better attitudes regarding SAP. The fact that physicians had better attitudes compared to nurses is not surprising, since SAP prescription is a medical duty. Differences by hospital may be explained by the presence of local guidelines, confirming that implementation of hospital protocols for antibiotic prophylaxis significantly enhances adherence to recommendations [28].

The study has a few limitations. It was conducted in three tertiary care children’s hospitals in Italy, and our results may not apply to other settings. In the qualitative study phase, participants to focus groups were not randomly sampled, and there might be a bias towards HCPs with a special interest in SAP. Nevertheless, the sampling approach in qualitative research is based on people’s multiple perspectives of the phenomena, including the full range of possible cases or settings so that the conceptual rather than statistical generalizations could be made [17]. In addition, some of the respondent’s meanings in the original language (Italian) might have been lost in the translation. In the quantitative phase, the survey response rate was >80%, but we cannot exclude that non respondents had different profiles and attitudes than HCPs who participated.

## Conclusions

Our study has identified several factors that may influence SAP in children, and need to be addressed to tailor quality improvement interventions.

Our findings support that a multifaceted intervention is needed to address HCPs barriers and improve attitudes. The intervention should include development of in-hospital policies for SAP, dissemination of performance and outcome indicators on adherence to recommendations and SSI incidence, and interactive education on SAP characteristics. Other settings may benefit from a similar analysis of barriers and determinants among HCPs, before developing sustainable and tailored interventions on SAP.

## Abbreviations

CI: Confidence Interval
GL: Guideline
HCP: Healthcare professional
N.: Number
OR: Odds Ratio
SAP: Surgical Antibiotic Prophylaxis
SSI: Surgical site infection
